# Promoter Hypermethylation Promotes the Binding of Transcription Factor NFATc1, Triggering Oncogenic Gene Activation in Pancreatic Cancer

**DOI:** 10.3390/cancers13184569

**Published:** 2021-09-11

**Authors:** Yenan Wu, Lea Kröller, Beiping Miao, Henning Boekhoff, Andrea S. Bauer, Markus W. Büchler, Thilo Hackert, Nathalia A. Giese, Jussi Taipale, Jörg D. Hoheisel

**Affiliations:** 1 Division of Functional Genome Analysis, German Cancer Research Center (DKFZ), Im Neuenheimer Feld 580, 69120 Heidelberg, Germany; wyn9191@aliyun.com (Y.W.); L.Kroeller@stud.uni-heidelberg.de (L.K.); b.miao@dkfz-heidelberg.de (B.M.); h.boekhoff@dkfz.de (H.B.); andrea.bauer@dkfz.de (A.S.B.); 2 Faculty of Biosciences, Heidelberg University, Im Neuenheimer Feld, 69120 Heidelberg, Germany; 3 Medical Faculty Heidelberg, Heidelberg University, Im Neuenheimer Feld, 69120 Heidelberg, Germany; 4 Department of General Surgery, University Hospital Heidelberg, Im Neuenheimer Feld 420, 69120 Heidelberg, Germany; markus.buechler@med.uni-heidelberg.de (M.W.B.); Thilo.Hackert@med.uni-heidelberg.de (T.H.); nathalia.giese@med.uni-heidelberg.de (N.A.G.); 5 Division of Functional Genomics, Department of Medical Biochemistry and Biophysics, Karolinska Institute, 171 65 Solna, Sweden; jussi.taipale@ki.se

**Keywords:** promoter methylation, transcription factors, gene regulation, pancreatic ductal adenocarcinoma

## Abstract

**Simple Summary:**

High promoter methylation is not necessarily associated with shutting down gene activity but does sometimes correlate with increased transcription instead. In a genome-wide analysis, we studied gene regulation in pancreatic cancer. We identified a substantial number of genes with both promoter hypermethylation and high transcription levels. Subsequently, we screened for transcription factors that exhibit specific binding to such hypermethylated sequences. NFATc1 was one of several transcription factors that bound specifically methylated DNA motifs and triggered transcription. A particularly affected gene was *ALDH1H3*, whose expression has strong oncogenic implications. Activation of *ALDH1H3* was due to a direct regulative process involving NFATc1 binding to its hypermethylated promoter. The results provide insights into the activation of gene transcription that is promoted by DNA methylation.

**Abstract:**

Studies have indicated that some genes involved in carcinogenesis are highly methylated in their promoter regions but nevertheless strongly transcribed. It has been proposed that transcription factors could bind specifically to methylated promoters and trigger transcription. We looked at this rather comprehensively for pancreatic ductal adenocarcinoma (PDAC) and studied some cases in more detail. Some 2% of regulated genes in PDAC exhibited higher transcription coupled to promoter hypermethylation in comparison to healthy tissue. Screening 661 transcription factors, several were found to bind specifically to methylated promoters, in particular molecules of the NFAT family. One of them—NFATc1—was substantially more strongly expressed in PDAC than control tissue and exhibited a strong oncogenic role. Functional studies combined with computational analyses allowed determining affected genes. A prominent one was gene *ALDH1A3*, which accelerates PDAC metastasis and correlates with a bad prognosis. Further studies confirmed the direct up-regulation of *ALDH1A3* transcription by NFATc1 promoter binding in a methylation-dependent process, providing insights into the oncogenic role of transcription activation in PDAC that is promoted by DNA methylation.

## 1. Introduction

The overall five-year survival rate for patients with pancreatic cancer is only 5 to 9%, and pancreatic tumours are basically considered incurable at current [1]. The carcinogenesis and development of pancreatic cancer are not only attributed to genetic alterations, but the important role of epigenetic regulation has become evident more recently. Toward a mechanistic understanding of how epigenetic processes regulate gene transcription, however, much further investigation is needed. As one type of epigenetic regulation, DNA methylation of the cytosine base normally occurs in cytosine-guanine dinucleotides (CpGs) [2]. Dysregulation of DNA methylation is considered as a hallmark of cancer and could aid in the stratification of cancer subtypes [3,4]. In pancreatic cancer, the promoters with the highest degree of DNA methylation were found in the genes *APC* (50% of cases), *BRCA1* (46%), *p16INK4a* (35%), *p15INK4b* (35%), *RARβ* (35%), and *p73* (33%). Further, in 94% of cases, methylation was observed in at least one of them [5]. For many genes associated with cancer a change in the degree of DNA methylation in the respective promoter is linked to transcriptional expression variations [6,7]. Particularly, an inverse correlation of the degree of DNA methylation and the level of gene expression has been reported [8]. Additionally, a set of 98 genes, which are silenced by DNA methylation in pancreatic cancer, were found to exert an influence on tumour development [9].

DNA methylation is generally thought to repress gene expression by obstructing the binding of transcription factors (TFs) to their binding sites and recruiting proteins with a methyl-CpG (mCpG)-binding domain (MBD) to compress the chromatin [10,11]. However, this traditional view has been challenged recently [12,13]. Some TFs without MBDs recognize methylated DNA motifs and affect biological function, such as gene expression [14], the recruitment of other TFs and related cofactors [15] and splicing regulation [16]. The methylated binding motifs of many TFs have been studied in a systematic manner. In one study, 47 TFs were found that could bind methylated sequences. Some of them recognized both the methylated and the non-methylated version of a binding motif [12]. A rather comprehensive investigation based on methyl-SELEX analysis revealed that CpG methylation influences the binding of most TFs. Particularly many developmentally important TFs (homeodomain, POU and NFAT proteins) seem to bind preferentially to mCpG sites [17]. Binding of some TFs and resulting transcription might actually be positively correlated with the methylation of their promoter recognition sites. This study aimed at identifying TFs, which exhibit specific binding to methylated promoter sequences in PDAC and thereby activate transcription that has oncological consequences. Deciphering methylation-dependent TF-promoter interactions and their roles in gene regulation and cellular function could provide further knowledge toward understanding PDAC biology and tumorigenesis.

## 2. Materials and Methods

### 2.1. Methylation Profiling

Genome-wide DNA methylation analysis was performed using the Infinium 450 k platform (Illumina, Munich, Germany) with DNA isolated from tissues of 26 PDAC patients and 24 healthy donors. The transcript profiles of the same samples had been studied earlier [18]. The samples were procured through the Pancobank at the EPZ/Surgery Department of the University of Heidelberg. In all cases, written informed consent had been obtained from the patients. The study was approved by the local ethics committee and performed in compliance with the provisions of the Declaration of Helsinki. In addition, the DNA of the tumour cell lines PANC-1 and MiaPaCa-2 was analysed. Data were processed using the default RnBeads workflow [19] and adjusted for multiple testing using Benjamini-Hochberg’s False Discovery Rate (FDR). Differences were selected which exhibited a *p*-value ≤ 0.05 and an absolute methylation difference ≥0.1.

### 2.2. Transcriptome Profiling

For analyzing transcript variations, cells were grown to a confluence of at least 50%, transfected with siRNA and incubated for another 40 h. RNA was extracted using the AllPrep DNA/RNA Mini Kit (Qiagen, Hilden, Germany) and analyzed on the Human HT12 Expression BeadChip (Illumina). The raw data was quantile normalized and log_2_ transformed. Expression analysis was performed using the LIMMA package and FDR-adjusted. Features with a *p*-value ≤ 0.01 and absolute log_2_ fold change (log_2_FC) ≥ 0.5 were considered significant. The data are accessible at the public repository ArrayExpress: http://www.ebi.ac.uk/arrayexpress/experiments/E-MTAB-10122 accessed on 9 September 2021; user: Reviewer_E-MTAB-10122; password: ukjgcesx).

### 2.3. Integration of Methylation and Expression Data

The average percentage of methylation was calculated for each promoter. Methylation changes were compared to expression variations of the respective genes. Gene ontology enrichment analysis was performed using the PANTHER14.1 classification system [20,21]. ‘GO biological process complete’ was chosen to be the annotation data set; the test type was Fisher’s exact. The threshold was set to an FDR-adjusted *p*-value of 0.05.

### 2.4. Microarray Screening

Protein microarrays were produced and incubated as described in full detail elsewhere [22,23,24]. Fluorescently labelled oligonucleotides representing both strands of the methylated or unmethylated *TWIST1* core promoter sequence (Hg38, chr7: 19,117,633–19,117,687; CpG dimers are underlined: AGTTGGGCGAGAGCTGCAGACTTGGAGGC TCTTATACCTCCGTGCAGGCGGAAAG; biomers.net, Ulm, Germany) were used. Image acquisition was on a Tecan scanner; feature extraction was done with GenePix Pro 6.0 (Molecular Devices, Sunnyvale, CA, USA). Median fluorescence intensities (MFI) were extracted. The mean MFI of negative controls plus four standard deviations was set as threshold for a positive signal.

### 2.5. Cell Culture

Pancreatic cancer cell lines PANC-1, BxPC-3, MiaPaCa-2, AsPC-1, Capan-1 and SUIT-2, as well as the epithelial cell line HPDE-E6E7, were purchased from ATCC and authenticated by the DKFZ Genomics Core Facility. All cells were regularly checked for mycoplasma contamination. PANC-1, BxPC-3 and Capan-1 were cultured in Iscove’s Modified Dulbecco’s Medium; MiaPaCa-2 and SUIT-2 were grown in Dulbecco’s Modified Eagle Medium (Thermo Fisher Scientific, Waltham, MA, USA). AsPC-1 was maintained in RPMI-1640 medium (Thermo Fisher Scientific), HPDE-E6E7 cells in Keratinocyte-SFM medium (Thermo Fisher Scientific). Culture media were supplemented with 10% FBS, 1% penicillin and 1% streptomycin (Thermo Fisher Scientific). Cell growth was at 37 °C, 5% CO_2_ and 95% humidity.

### 2.6. siRNA Gene Knockdown

Gene knockdown was performed using anti-*NFATc1* MISSION pre-designed siRNA molecules (Sigma-Aldrich, Taufkirchen, Germany) with Lipofectamine 2000 (Invitrogen, Darmstadt, Germany) following the manufacturer’s instructions. As control, siRNA of scrambled sequence (Santa Cruz Biotechnology, Dallas, TX, USA) was used. Cells were harvested 24 h post transfection for real-time qPCR. Gene set enrichment analysis was performed using the Broad Institute algorithm [25]. Gene sets were considered as significantly enriched whose Normalized Enrichment Score (NES) was above 1 with a *p*-value ≤ 0.05 and FDR *q*-value ≤ 0.25.

### 2.7. CRISPR/Cas9-Mediated Knockout

sgRNA targeting *NFATc1* was synthesized by biomers.net (Table S1) and cloned into the lentiviral vector pL-CRISPR.EFS.GFP. For lentivirus production, HEK-293T cells were used [26]. Lentivirus supernatant was harvested after two days of cell growth. For lentivirus transduction, cells were seeded in 6-well plates (2.5 × 10^5^ cells each) and grown to 70–80% confluency. Positively transduced cells were isolated by cell sorting (FACSAria III; BD Biosciences, Heidelberg, Germany) using the vector’s GFP signal.

### 2.8. Overexpression of NFATc1

For stable NFATc1-overexpressing cells, the *NFATc1* ORF was cloned into overexpression vector pCDH (System Biosciences, Palo Alto, CA, USA). Correct cloning was verified by DNA sequencing. After lentiviral transduction into PANC-1 and MiaPaCa-2, transduced cells were isolated by cell sorting and expanded.

### 2.9. Quantitative Real-Time PCR

RNA extraction was performed using the AllPrep DNA/RNA mini kit (Qiagen). cDNA synthesis was performed with 1 µg of total RNA using the ProtoScript First Strand cDNA Synthesis kit (New England Biolabs, Frankfurt, Germany). Quantitative real-time PCR was done using the Fast SYBR Green qPCR kit (Thermo Fisher Scientific) on a LightCycler 480 (Roche Diagnostics, Mannheim, Germany); *HPRT1* was used as housekeeping control. The data was analyzed with the ΔΔCt method. All qPCR primers (*HPRT1*, *GAPDH*, *ALDH1A3*, *MKNK2*, *SLC7A5*) were purchased from Qiagen (Quantitect primer assay).

### 2.10. Western Blot Analysis

For protein extraction, RIPA Lysis and Extraction buffer (Thermo Fisher Scientific), supplemented with Halt Protease and Phosphatase Inhibitor Cocktail, phenylmethylsulfonylfluoride (PMSF) and Benzonase Nuclease (Merck, Darmstadt, Germany) was used according to the manufacturer’s protocol. Fifteen micrograms protein were resolved by gel electrophoresis and blotted to nitrocellulose using a Trans-Blot Turbo System (Bio-Rad, Feldkirchen, Germany). Membranes were blocked with 5% non-fat milk and incubated with anti-NFATc1 antibody (7A6; Thermo Fisher Scientific) or anti-GAPDH antibody (Sigma-Aldrich) overnight at 4 °C. After incubation with the corresponding secondary antibody (anti-mouse or anti-rabbit IgG (H+L) peroxidase; Vector Laboratories, Burlingame, CA, USA), blots were developed by means of ECL Western blot substrate (Merck). Images were acquired with a luminescence imager LAS 3000 mini (FujiFilm, Düsseldorf, Germany).

### 2.11. Migration Assay

Cell migration assays were performed in 24-well plates with TC inserts of 8 µm pore size (Sarstedt, Nümbrecht, Germany) 24 h after *NFATc1*-specific siRNA knockdown. To each insert, 50,000 cells in 0.2 mL FBS-free medium were added; 0.5 mL chemo-attractant (10% FBS complete culture medium) was added to the well. A 24 h incubation was followed by crystal violet staining. An Axio Examiner Z1 microscope (Zeiss, Oberkochen, Germany) was used for taking pictures. Five different fields of view were captured. ImageJ (version 153f, National Institute of Mental Health, Bethesda, MD, USA) was used to measure the area of crystal violet staining.

### 2.12. Colony Assay

In each well of 6-well plates, 2 mL 0.5% soft SeaKem GTG agarose (Biozym, Oldendorf, Germany) was plated. Then, 3300 cells were resuspended in 1 mL 0.05% agar and put on top. After 20 min, 1 mL culture medium was added. Incubation was for three weeks. Subsequently, 0.5 mL of 0.005% crystal violet was added to each well and shaken for more than 1 h. After washing with PBS, images were taken. OpenCFU-3.8 BETA software (SourceForge, San Diego, CA, USA) was used for image analysis.

### 2.13. Demethylation Treatment, DNA Bisulfite Conversion and Sequencing

We performed initial analyses with different 5-azacytidine (Sigma-Aldrich) concentrations (0–10 µM) and varying incubation times (0–4 days) to optimise the demethylation effect without harming the cells by the treatment. Eventually, PANC-1 or MiaPaCa-2 cells were grown in presence or absence of 2 μM or 1 µM 5-azacytidine, respectively; growth medium was replaced every 24 h. After 72 h, genomic DNA was isolated (AllPrep DNA/RNA Mini Kit; Qiagen); 1 μg DNA was used for bisulfite conversion with the EpiTect Bisulfite Kit (Qiagen). The region of interest was amplified from this DNA (Table S1) and cloned into pCR2.1 vector using a TA Cloning kit (Thermo Fisher Scientific). Sanger sequencing was performed by Eurofins (Ebersberg, Germany). Sequences were aligned with the program BiQ analyzer [27]. The conversion rate was determined on the basis of sequencing ten independent copies of each fragment.

### 2.14. Luciferase Assay

The *ALDH1A3* promoter region (−1035/−893; hg19, chr15:101,418,974–101,419,116) was PCR-amplified (Table S1). The purified product was cloned into the luciferase reporter plasmid pCpGL-basic [28]. The construct without the *ALDH1A3* promoter was used as control. PANC-1 and MiaPaCa-2 cells were grown for 24 h and then transfected with the luciferase constructs. After 24 h, cells were harvested and the luciferase activity was measured using the Dual-Luciferase Reporter Assay kit (Promega, Medison, WI, USA).

### 2.15. Chromatin Immunoprecipitation (ChIP)

ChIP was performed using the SimpleChIP Enzymatic Chromatin IP Kit (Cell Signaling Technology, Danvers, MA, USA). Five micrograms of anti-NFATc1 antibody (7A6; Thermo Fisher Scientific) was used; a complex, unspecific IgG mixture (Vector Laboratories) acted as control. Sequence enrichment was determined by qPCR on a LightCycler 480 (Roche Diagnostics) (Table S1). Three replicate analyses were carried out for each sample. The percent input method was used for normalization.

## 3. Results

### 3.1. Identification of Genes with Changes in Both Promoter Methylation and RNA Expression

**Figure 1:**
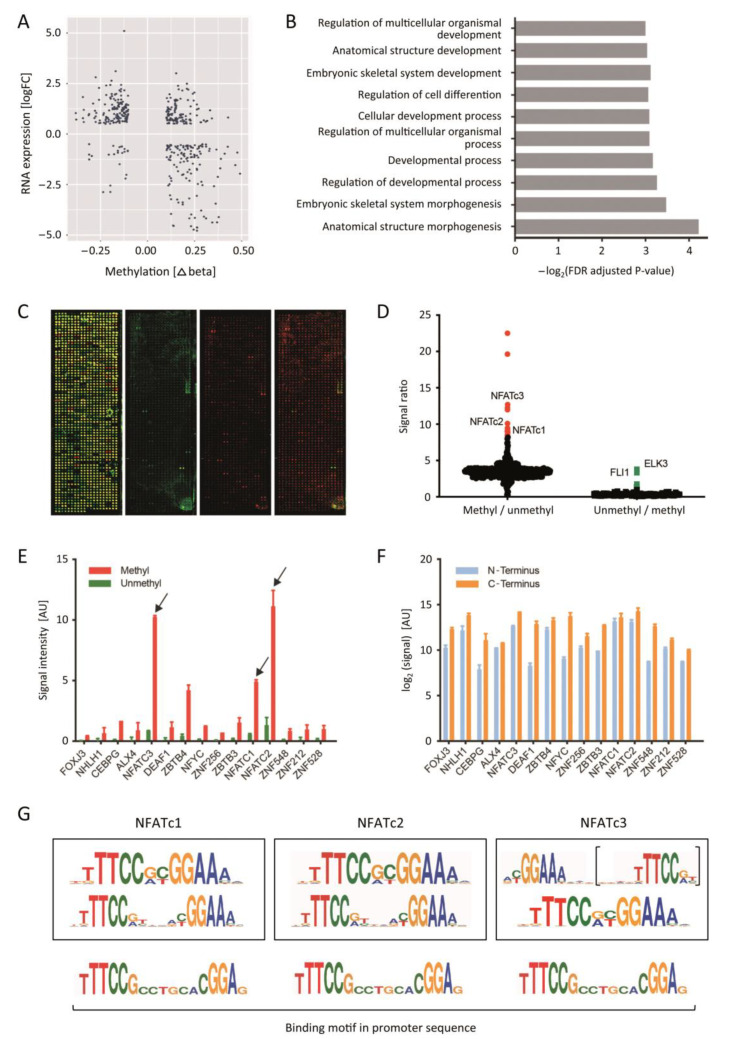
Identification of transcription factors that preferentially bind to methylated sequences. (**A**) Integration of expression and methylation profiling data. Along the x-axis, DNA methylation variations between PDAC and healthy tissues are shown as the mean delta beta value in the promoter regions (−1500/+500 bp); the log-fold change of transcript levels is presented along the y-axis. Each dot represents one gene. (**B**) Gene ontology term enrichment analysis of up-regulated and hypermethylated genes. The top 10 overrepresented processes are shown. (**C**) Typical images of the protein microarray with 658 TF DNA binding domains and seven full-length TFs. Left: Terminal tags present in each protein were stained with fluorescently labelled antibodies. Right: Results of incubations with an unmethylated (green) and methylated (red) 55 bp DNA fragment resembling the *TWIST1* promoter; beside the individual images, also a merger is shown at the far right. (**D**) Signal ratios were calculated of methylated over unmethylated and vice versa signal intensities. Red or green dots highlight TF candidates, which preferably bound the methylated or unmethylated DNA fragment, respectively. (**E**) The signal intensities of the top 15 TFs with preferential binding to the methylated sequence is shown; NFATc1, NFATc2 and NFATc3 as well as ZBTB4 exhibited the strongest binding by far. (**F**) As a control, the relative protein expression level of these TFs was deduced from the antibody labelling of their terminal tags. (**G**) The methylated recognition motifs of NFATc1, NFATc2 and NFATc3 as extracted from MeDReaders [29] are depicted (boxes) in comparison to a binding motif identified in the microarray screen.

mRNA expression profiling data of 195 PDAC and 41 healthy pancreas tissues were available from previous studies [18]. These profiles were integrated with whole-genome methylation information produced with the Illumina microarray assay that covers 450,000 genomic methylation sites. Genomic DNA isolated from tumour tissues was analysed which had been subjected to transcriptional profiling. The methylation values of CpG sites were averaged across all PDAC and healthy pancreas samples, respectively. Comparing PDAC versus healthy pancreas, 420 genes showed dysregulated RNA expression along with a change in promoter methylation (Table S2). Of these, 119 genes exhibited both up-regulated RNA expression and promoter hypermethylation (Figure 1A; Table S2). This amounts to more than a quarter of the genes that showed alterations in both methylation and RNA expression. Only 23 genes displayed the inverse result of lower promoter methylation and a simultaneous decrease of transcript level in PDAC. The 119 genes with high methylation and stronger RNA expression were used as input for a gene ontology term enrichment analysis to find possible functional roles. The 10 most overrepresented functional processes are all associated with embryonic development (Figure 1B). A similar analysis with the 23 low methylation and low expression genes did not yield any significant result.

### 3.2. The NFAT TF-Family Binds to DNA in a Methylation-Dependent Differential Manner

In order to study methylation as a transcription regulation mechanism, we investigated the interaction of TFs with methylated and unmethylated DNA fragments representing promoter sequences. To this end, we produced a protein microarray presenting the binding domains of 658 TFs and seven full-length TFs (Table S3); the latter were mainly added to compare the binding behaviour of the respective binding domain and the complete TF. Array production was by enzymatic in situ protein synthesis [22]. Protein expression was assessed by staining with fluorescently labelled antibodies against a 6His-tag and a V5-tag that were introduced to the N- and C-terminus of each protein, respectively (Figure 1C). At least 97% of the TFs were successfully expressed at full length. Several known consensus DNA sequences were used for validating protein functionality, confirming the appropriate functioning and specific binding of the arrayed TFs [23].

The promoter of gene *TWIST1* (hg19, chr7:19,117,633–19,117,687) was of particular interest as it exhibited in our data the most hypermethylated CpG sites comparing PDAC to healthy tissue samples. Synthetic, fluorescently labelled DNA-fragments of the sequence were synthesized that represented either the fully methylated or the completely unmethylated promoter fragment. Using equimolar amounts, we incubated the fragments on TF microarrays for the identification of candidate binders (Figure 1C). Few TFs showed preferential binding to the unmethylated sequence (e.g., FLI1 and ELK3) (Figure 1D), while others did not discriminate between methylated or unmethylated sequence, such as HOXA7 or ZNF655. Within the 15 TFs that exhibited the highest preference for the methylated sequence, NFATc1, NFATc2 and NFATc3 were the TFs that showed the strongest binding (Figure 1E). All three belong to the Nuclear Factor of Activated T-Cells (NFAT) family. In order to avoid that protein expression levels might have caused a strong bias, the expression of each candidate was checked. There was full-length protein expression in all cases and the protein amounts were comparable (Figure 1F). A part of the promoter sequence applied to the TF microarray exhibited high similarity to the predicted methylated sequence binding motifs determined for NFATc1, NFATc2 and NFATc3 [17] (Figure 1G) including two CpG sites. Beside TFs of the NFAT family, also other molecules, such as POU homeodomain TFs, were found to bind preferentially to methylated DNA.

### 3.3. NFATc1 Is Playing an Oncogenic Role

**Figure 2:**
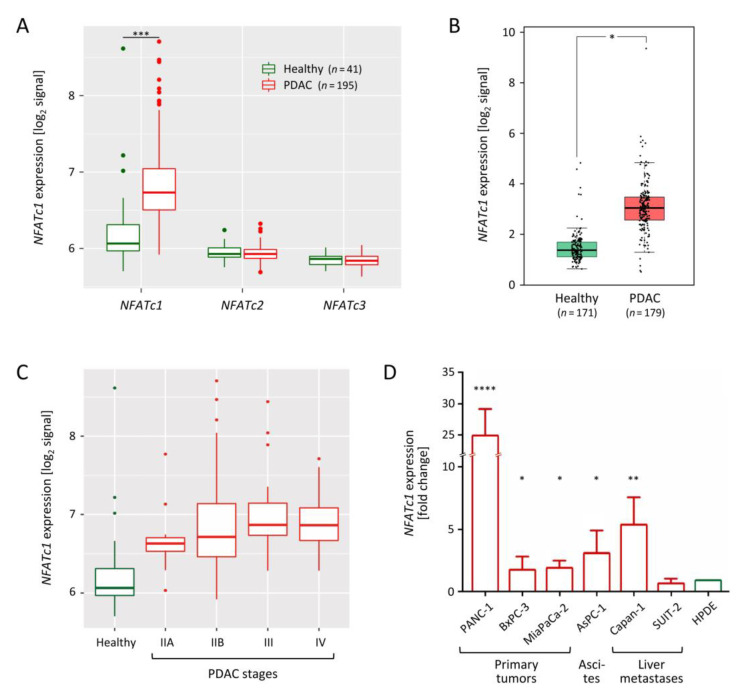
Expression of *NFATc1* in pancreatic cancer cell lines and tissues. (**A**) mRNA levels of NFAT TF genes in PDAC and healthy tissues [18]. (**B**) Same analysis for *NFATc1* on the basis of data from the TCGA and GTEx data repositories. (**C**) Variation in *NFATc1* transcript levels across PDAC stages. (**D**) Quantification of *NFATc1* mRNA levels in tumor cell lines in comparison to the non-cancer cell line HPDE-E6E7 (HPDE). * = *p* ≤ 0.05; ** = *p* ≤ 0.01; *** = *p* ≤ 0.001; **** = *p* ≤ 0.0001.

Expression of *NFATc1*, *NFATc2* and *NFATc3* was analysed in the tissue RNA profiling data. Only the transcript level of *NFATc1* was found to be up-regulated in PDAC tissues relative to samples from healthy donors (Figure 2A). For confirmation, we also took advantage of datasets available at The Cancer Genome Atlas (TCGA) and Genotype-Tissue Expression (GTEx) data repositories. These datasets were in full agreement confirming the up-regulation of *NFATc1* in a tumour (Figure 2B). Transcript levels did not change significantly across PDAC stages but remained high as compared to the level in healthy pancreas tissue (Figure 2C). The mRNA expression of *NFATc1* was also explored in the six PDAC cell lines PANC-1, BxPC-3, MiaPaCa-2, AsPC1, Capan-1 and SUIT-2 in comparison to the non-cancer immortalized human pancreatic duct epithelial cell line HPDE-E6E7 [30]. Quantitative PCR showed that *NFATc1* was up-regulated in all cancer cell lines apart from SUIT-2; the effect was strongest in PANC-1 (Figure 2D).

**Figure 3:**
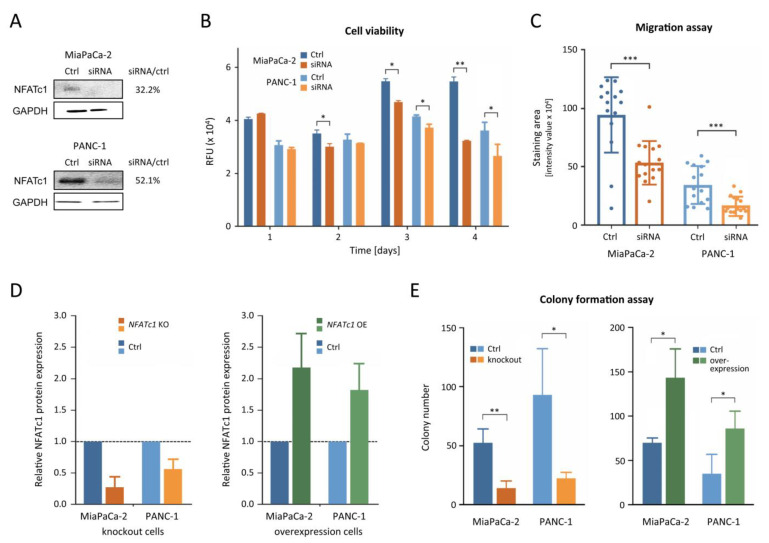
Functional analysis of NFATc1. (**A**) Typical Western blot of NFATc1 protein levels in cell lines PANC-1 and MiaPaCa-2 upon gene knockdown (siRNA) in comparison to controls with constructs of scrambled sequence; the normalized ratios are given. (**B**) Viability of *NFATc1*-knockdown (siRNA) or control cells over a period of four days. (**C**) For the same cells, the migration ability was determined after two days. (**D**) Normalized signal intensities of protein NFATC1 on Western blots from cells with CRISPR/Cas-mediated *NFATC1* knockout (KO) or overexpression (OE), respectively, in comparison to cells transfected with an sgRNA of unspecific, scrambled sequence (Ctrl; 100% level). (**E**) The colony formation capacity of these cells was analysed. * = *p* ≤ 0.05; ** = *p* ≤ 0.01; *** = *p* ≤ 0.001. The raw and normalized signal intensity values and images of the original blots of panels A and D are presented in Table S4.

Since NFATc1 was among the TFs with strongest binding to the methylated promoter and also up-regulated in PDAC tissues, it was looked at in further detail. To this end, siRNA-mediated knockdown cell models were created of PANC-1 and MiaPaCa-2. Western blot analysis confirmed the knockdown of *NFATc1* (Figure 3A). In both models, down-regulation of *NFATc1* decreased cell viability significantly. While the effect was small initially and not detectable after one day, it accumulated over time and led to significant differences in both models after three days (Figure 3B). Intriguingly, the effect was seen first in the cell line MiaPaCa-2 with the less pronounced up-regulation compared to HPDE-E6E7. Cell migration was also tested after two days of knockdown. A reduction in NFATc1 significantly suppressed cell migration in both PANC-1 and MiaPaCa-2 (Figure 3C). For further characterization by a colony formation assay, stable knockout, and overexpression variants of *NFATc1* were produced in PANC-1 and MiaPaCa-2 (Figure 3D). In both cells, colony formation was strongly decreased upon knockout of *NFATc1* (Figure 3E) while, in contrast, the cells overexpressing NFATc1 formed more than twice as many colonies as the control. In summary, viability, migration, and colony formation assays revealed that NFATc1 is playing an oncogenic role in pancreatic cancer cell lines.

### 3.4. Identification of ALDH1A3 as a Target Gene of NFATc1

**Figure 4:**
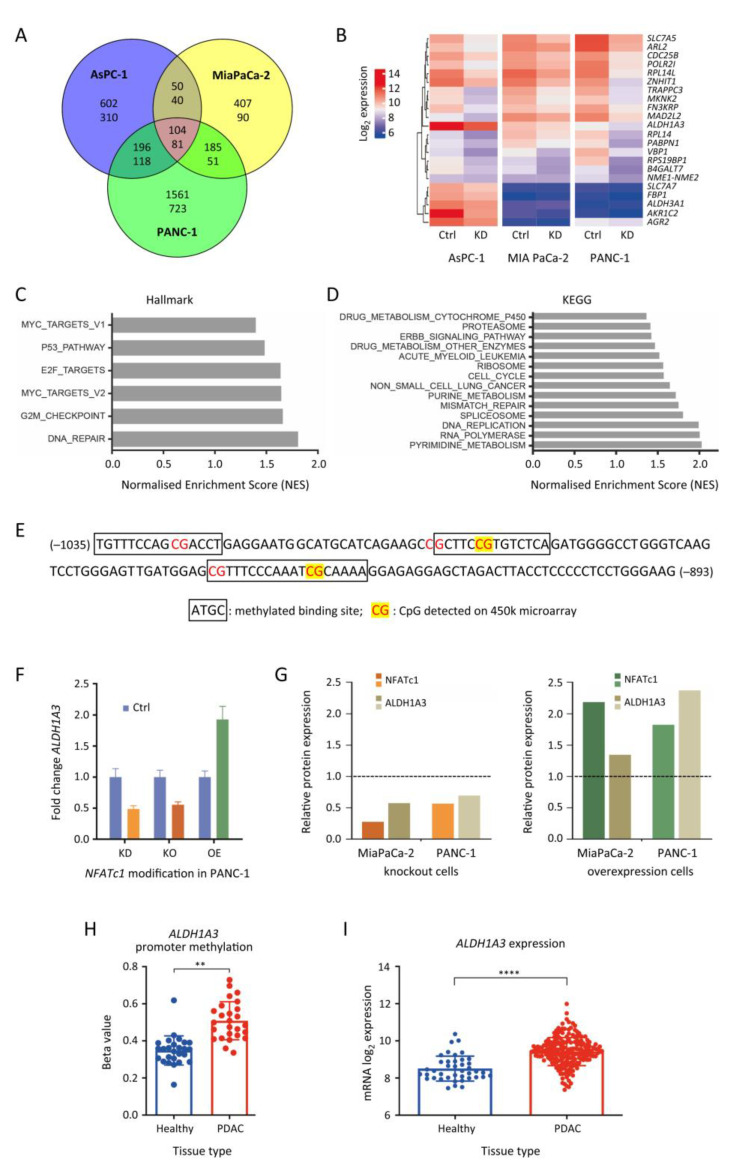
Analysis of NFATc1-based gene regulation. (**A**) Number of genes with significant transcript level variations in three cell lines transfected with *NFATc1* siRNA compared to cells transfected with a control siRNA of unspecific, scrambled sequence. The upper and lower numbers give the number of genes with higher or lower transcript levels in the knockdown cells, respectively. (**B**) Heatmap showing the top 24 genes of the total of 81 genes that were consistently downregulated in all three cell lines upon *NFATc1* knockdown. Results of a hallmark (**C**) and KEGG gene set enrichment analysis (**D**) based on the cell line *NFATc1* knockdown transcript profiling. The normalized enrichment score (NES) was used to compare analysis results across gene sets. (**E**) Identification of methylated NFATc1 binding sites in the promoter region of *ALDH1A3* using the FIMO tool of The MEME Suit [31]; binding sites are framed; CpG sites are coloured in red. (**F**) Changes in *ALDH1A3* expression determined by qPCR upon knockdown (KD), knockout (KO) or overexpression (OE) of the *NFATc1* gene in PANC-1. (**G**) The change of ALDH1A3 protein expression upon KO and OE of *NFATc1* was also determined. Promoter methylation (**H**) and mRNA expression (**I**) of *ALDH1A3* in tissues from PDAC patients or healthy donors. ** = *p* ≤ 0.01; **** = *p* ≤ 0.0001.

In order to understand the genome-wide reactions at transcript level resulting from knocking down *NFATc1*, we performed transcriptome profiling in the pancreatic cancer cell lines PANC-1, MiaPaCa-2 and AsPC1. Cells transfected with *NFATc1* siRNA were compared to cells transfected with a control siRNA of unspecific, scrambled sequence. We looked for significant changes that happened in all three cell lines. *NFATc1* knockdown consistently triggered lower transcript levels of 81 genes (Figure 4A,B). Gene set enrichment analysis [25] was performed on the basis of the transcript profiling data so as to get some functional leads. When the analysis was performed in reference to the MSigDB hallmark gene sets, genes associated with Myc targets, the P53 pathway, E2F targets, G2M checkpoint, and DNA repair were significantly enriched in the control tumour cells compared to the *NFATc1*-knockdown cells (Figure 4C). An analysis related to the KEGG pathways (Figure 4D) also revealed enrichment in genes that belong to cancer-associated pathways in the tumour cells, compared to their *NFATc1-* knockdown equivalent, including several associations with metabolism. Metabolism reprogramming is linked to cancer initiation, metastasis formation and tumour recurrence.

Within the group of genes that consistently showed the strongest down-regulation in knockdown cells (Figure 4B), three genes—*ALDH1A3, ALDH3A1* and *AGR2*—were hypermethylated in their promoter regions in PDAC tissues compared to healthy tissues. The promoter sequences of these three genes were analysed with the FIMO tool of the MEME Suit [31] in order to identify binding motifs of NFATc1. Several methylated binding sites were found in the promoter region of *ALDH1A3* (Figure 4E). Aldehyde dehydrogenase family 1 member A3 (ALDH1A3) is one of the most important aldehyde metabolic enzymes, fitting functionally to the predictions made on the basis of the enrichment analyses. Its expression is associated with the development, progression, and prognosis of cancers and it acts as a marker for cancer stem cells [32]. For validating the *ALDH1A3* promoter as a target of NFATc1, *NFATc1*-knockdown, knockout, and overexpression cell models were used to study *ALDH1A3* mRNA levels (Figure 4F). *ALDH1A3* was suppressed in cells, in which NFATc1 was depleted, and up-regulated upon NFATc1 overexpression. We also looked at the ALDH1A3 protein expression. Consistent with knockout or overexpression of *NFATc1*, the level of ALDH1A3 changed accordingly (Figure 4G). In addition, PDAC tissue samples were studied. In comparison to healthy tissue, *ALDH1A3* was both hypermethylated in the promoter region (Figure 4H) and up-regulated (Figure 4I) in PDAC.

### 3.5. NFATc1 Regulates ALDH1A3 in a Methylation-Dependent Way

**Figure 5:**
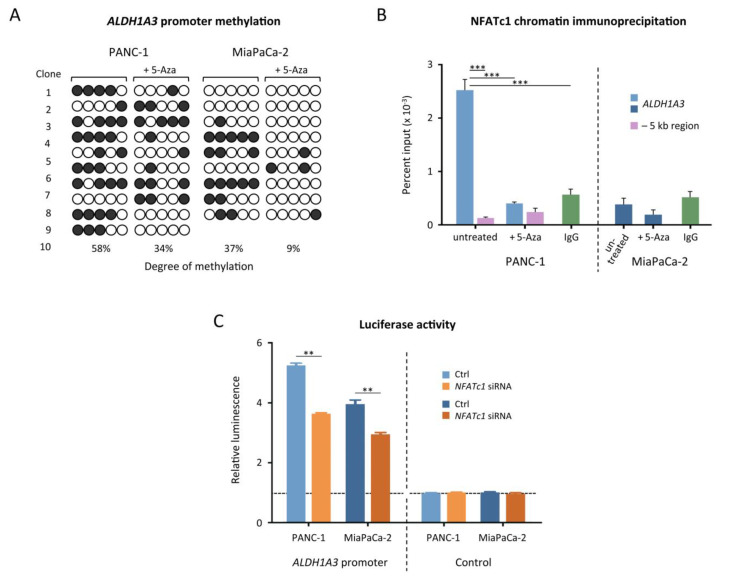
Methylation-mediated transcription regulation. (**A**) Bisulphite-sequencing result of the *ALDH1A3* promoter (−1035 bp/−893 bp), in PANC-1 and MiaPaCa-2 before and after a treatment with 5-azacytidine (5-Aza). Each circle corresponds to a CpG site in this region, each line refers to one cloned allele. Filled circles represent methylated cytosines, empty circles stand for unmethylated cytosines. (**B**) Chromatin immunoprecipitation with antibody targeting NFATc1. The enrichment of the *ALDH1A3* promoter region is shown in comparison to an arbitrarily selected genomic region located 5 kb upstream of the promoter (−5 kb) and results obtained with an IgG mix instead of NFATc1-specific antibody (IgG). Results after treatment with 5-Aza are also shown. (**C**) Relative luciferase activity in PANC-1 and MiaPaCa-2 cell lines. The *ALDH1A3* promoter sequence was cloned into a vector construct encoding the luciferase gene. After transfection, luminescence was measured as an indicator of promoter activity. In accordance with the methylation level, higher signal was obtained in PANC-1 than MiaPaCa-2. A knockdown of *NFATc1* (siRNA) also resulted in reduced signal intensities. For comparison, the empty vector was used as a control. ** = *p* ≤ 0.01; *** = *p* ≤ 0.001.

For confirming the actual binding of NFATc1 to the methylated *ALDH1A3* promoter, we performed chromatin immunoprecipitation (ChIP) in PANC-1 and MiaPaCa-2. Besides growing the cells under standard conditions, we also treated the two cell lines with the demethylation agent 5-azacytidine. In all experiments, genomic DNA was isolated and subjected to bisulphite sequencing in order to determine the actual methylation level of the NFATc1 binding sites in the *ALDH1A3* promoter. The five CpG sites exhibited an average methylation level of 58% in PANC-1 and 34% in MiaPaCa-2 (Figure 5A). After treating the cells with 5-azacytidine, the average methylation significantly decreased to 37% in PANC-1 and 9% in MiaPaCa-2. In the ChIP analysis with NFATc1-specific antibody, quantitative PCR was employed for detecting sequence enrichment. As a control, an arbitrarily selected, unrelated DNA segment located 5 kb upstream of the *ALDH1A3* promoter was used. Significant enrichment of the *ALDH1A3* promoter sequence was observed in PANC-1 (Figure 5B). However, only a background level signal was produced upon treatment of the PANC-1 cells with 5-azacytidine. It was comparable to that of the unspecific control region that was 5 kb upstream and less than that of another control, in which a complex mixture of rabbit IgGs isolated from pooled rabbit sera was used in the immunoprecipitation rather than an NFATc1-specific antibody. Given the similar level of methylation of PANC-1 after treatment with 5-azacytidine and untreated MiaPaCa-2 and in consideration of the fact that less NFATc1 is expressed in MiaPaCa-2, it is not surprising that no enrichment could be observed with MiaPaCa-2 (Figure 5B).

A luciferase assay was performed to further document the activating effect of DNA methylation and binding of NFATc1 on gene transcription. The *ALDH1A3* promoter region was cloned into a construct with the luciferase reporter gene, which was then brought into both PANC-1 and MiaPaca2. PANC-1 cells showed an overall higher promoter activity (Figure 5C) indicated by the stronger luminescence signal produced in these cells; this correlates with the higher degree of methylation in PANC-1 compared to MiaPaCa-2. Knocking down *NFATc1* clearly reduced the luminescence signal in both cell lines. In all cases, the promoter activity was higher compared to that of a control construct without the *ALDH1A3* promoter region. The results demonstrate the combined effect of three factors on transcriptional activity: the existence of NFATc1 binding sites in the promoter, the presence of the transcription factor itself, as well as the degree of promoter sequence methylation.

## 4. Discussion

DNA methylation is a key epigenetic modification and an important regulative factor in many cancers, including PDAC [33]. Methylation of promoter sequences usually leads to a more densely packed chromatin [34], negatively affecting transcription This process can be explained by the classical model that proteins with MBDs bind to methylated sequences specifically and recruit repressor complexes, such as a histone deacetylase complex. The histone deacetylation is responsible for a condensed chromatin structure, which then blocks transcription [35]. This model does not explain, however, how increased rather than reduced expression could be mediated by promoter hypermethylation. More recent evidence suggests a rather competitive scenario of methylation dependent transcription regulation, in which methylated sequences could also attract the binding of TFs that specifically recognize methylated binding motifs and thus initiate transcription [13,29].

In order to gain some insight about the scale of coinciding promoter hypermethylation and transcript activation in PDAC, we analysed globally in the very same sample set the variations in the methylation of the genomic DNA and the abundance of mRNA. Altogether, 420 genes showed a change in both transcription and the degree of promoter methylation. This is 8% of the overall 5196 genes that exhibited transcriptional changes [18]. Slightly more than a quarter of these genes combined higher promoter methylation with higher transcript levels. Apparently, only a minority of the overall changes in transcriptional activity in PDAC seems to depend on variations of promoter methylation and only some 2% exhibit higher transcription coupled to promoter hypermethylation.

Toward a better understanding of the influence of DNA methylation as a mechanism of transcription regulation, we looked for TFs that bind preferentially to methylated promoter regions. The promoter sequence of *TWIST1* was an obvious choice for this analysis, since *Twist1* is one of the genes with higher expression in PDAC combined with a hypermethylation in its promoter. Actually, it had exhibited the highest degree of promoter sequence methylation in our analysis. Overexpression of *Twist1* and high methylation of its promoter have frequently been found in metastatic carcinomas [36]. Functionally, it boosts epithelial-mesenchymal transition, and increases both cell migration and invasion. *Twist1* has also been shown to be involved in the evasion of apoptosis and acts as a marker for cancer stem cells [37]. In addition, it affects the glucose metabolism in PDAC, promoting proliferation [38]. However, also results that oppose the findings in PDAC and other tumours have been reported. In lung cancer, high promoter methylation is actually correlated with low *Twist1* expression, for example [36]. This indicates a variable regulative functioning of DNA methylation in conjunction with different TFs. This assumption is further corroborated by the fact that *TWIST1* was not found to be regulated in PDAC cell lines by knocking down *NFATc1*. In combination with the result that several TFs were able to bind the methylated promoter sequence, this suggests that not merely the methylation and the presence of a binding motif in the promoter are regulating gene expression, but further factors are involved.

In order to understand the mechanism of oncogenic activation further, information about the *NFATc1*-associated pathways and potential targets of NFATc1 binding were combined with data from gene knockdown experiments and methylation profiling of PDAC tissues. *ALDH1A3* was identified as a prime target of NFATc1 and confirmed as being directly and positively regulated by the degree of promoter methylation and NFATc1 concentration. Its activation had strong and immediate oncogenic consequences. NFATc1 is known to promote tumour progress [39,40]. In colon and pancreatic cancer, NFATc1 promotes cell growth by inducing the expression of c-Myc and cyclin-D [41,42]. Increased NFATc1 expression has also be reported to cause acinar cell trans-differentiation, initiating pancreatic cancer [43]. Moreover, histone methyltransferase EZH2 upregulates NFATc1, which is important for pancreatic cell plasticity [44]. NFATc1 interacts with CBP/p300 via its N-terminal transactivation domain (TAD) [45], which transfers acetyl groups from acetyl-CoA to histones. This results in chromatin relaxation and the recruitment of the transcriptional machinery [46]. While the members of the NFAT TF-family share conserved domains, the TAD regions are variable and may have a critical role in the different functioning of the respective TFs.

## 5. Conclusions

With respect to methylation-dependent activation of transcription, NFATc1 recognizes and binds to the methylated promoter sequence, serving as an anchor. CBP/p300 could then interact with the TAD of NFATc1 and acetylate the histone, thus affecting the chromatin structure and activate transcription. More information about NFATc1-in-duced chromatin modification and the advent of co-activators is needed to confirm this scenario.

## Data Availability

The cell line transcriptional profiling data is accessible at the public repository ArrayExpress (ID: Reviewer_E-MTAB-10122; password: ukjgcesx).

## Supplementary Materials

The following are available online at https://www.mdpi.com/article/10.3390/cancers13184569/s1, Table S1: Oligonucleotide sequences, Table S2: Genes exhibiting a change (up or down) in both promoter methylation and transcript level, Table S3: List of transcription factors, Table S4: Western blot quantification.
