# Supplementary material for: Promoter Hypermethylation Promotes the Binding of Transcription Factor NFATc1, Triggering Oncogenic Gene Activation in Pancreatic Cancer
Source: Cancers (Basel). 2021 Sep 11;13(18):4569. doi: 10.3390/cancers13184569 (PMC8471171; doi:10.3390/cancers13184569)
Supplement: Supplementary file 1 [file cancers-13-04569-s001.zip › Supplementary Table S1.pdf]

Supplementary Table 1. Oligonucleotide sequences.

Oligomer name	Sequence (5'-3')	Note
NFATc1_sg2_1	CACCGCGGAGGACACCCCATCGTGC	sgRNA sequence
NFATc1_sg2_2	AAACGCACGATGGGGTGTCCTCCGC	
Scramble_1	CACCGATATCCGGAATTCGCGCGAT	sgRNA sequence
Scramble_2	AAACATCGCGCGAATTCCGGATATC	
Bisulfite_seq_for	GGAAAGAGTTGGTATTGTTTAGATT	Bisulfite sanger sequencing
Bisulfite_seq_rev	AACACATAAAACCTTTACTACTAAT	
Luciferase_for	CACAGATCTTGTTCCAGCGACCTGAGGAAT	Promoter cloning into luciferase vector
Luciferase_rev	ATTCCATGGCTTCCCAGGAGGGGGAGGTAA	
ALDH1A3_ChIP_for	TCGCCAGTGTTAGCCAGCCGATAT	ChIP PCR
ALDH1A3_ChIP_rev	AAAGGTCTTGTGCTGTTATGGCCT	
