# Supplementary material for: Promoter Hypermethylation Promotes the Binding of Transcription Factor NFATc1, Triggering Oncogenic Gene Activation in Pancreatic Cancer
Source: Cancers (Basel). 2021 Sep 11;13(18):4569. doi: 10.3390/cancers13184569 (PMC8471171; doi:10.3390/cancers13184569)

Table S4. Quantitative measurements of the signal intensities obtained from the Western blots. The data shown in Figure 3A,D and Figure 4G represent the normalized ratios of the results from affected cells and the respective controls. Further below, the cropped and uncropped images of the actual Western blots are shown.

Knockdown with siRNA:

**Analysis**	**Protein**	**Signal Intensity [Arbitrary Units]**		**Normalized to 100,000 Signal Units GAPDH**	**Ratio % siRNA/Control**
siRNA	NFATC1	MiaPaCa-2 NFAT control	264,708	231,069	**32.2**
		MiaPaCa-2 NFAT siRNA	88,412	74,354	
	GAPDH	MiaPaCa-2 NFAT control	114,558		
		MiaPaCa-2 NFAT siRNA	118,907		
	NFATC1	PANC-1 NFAT control	332,993	386,137	**52.1**
		PANC-1 NFAT siRNA	162,180	201,361	
	GAPDH	PANC-1 NFAT control	86,237		
		PANC-1 NFAT siRNA	80,542		

Knockout results:

Analysis	Protein	Signal Intensity [Arbitrary Units]		Normalized to 100,000 signal units GAPDH	Ratio % KO / control
Knockout (KO)	NFATC1	MiaPaCa-2 NFATC1 control	195,748	151,648	**43.7**
		MiaPaCa-2 NFATC1 KO	76,851	66,317	
	GAPDH	MiaPaCa-2 NFATC1 control	129,080		
		MiaPaCa-2 NFATC1 KO	115,885		
	NFATC1	PANC-1 NFATC1 control	257,411	176,781	**40.4**
		PANC-1 NFATC1 KO	111,277	71,434	
	GAPDH	PANC-1 NFATC1 control	145,610		
		PANC-1 NFATC1 KO	155,777		
Knockout (KO)	NFATC1	MiaPaCa-2 NFATC1 control	159,469	269,460	**10.6**
		MiaPaCa-2 NFATC1 KO	36,809	28,447	
	GAPDH	MiaPaCa-2 NFATC1 control	59,181		
		MiaPaCa-2 NFATC1 KO	129,397		
	NFATC1	PANC-1 NFATC1 control	92,849	110,212	**70.3**
		PANC-1 NFATC1 KO	64,506	77,465	
	GAPDH	PANC-1 NFATC1 control	84,246		
		PANC-1 NFATC1 KO	83,271		
Knockout (KO)	ALDH1A3	MiaPaCa-2 NFATC1 control	58,175	98,300	**57.6**
		MiaPaCa-2 NFATC1 KO	73,294	56,643	
	GAPDH	MiaPaCa-2 NFATC1 control	59,181		
		MiaPaCa-2 NFATC1 KO	129,397		
	ALDH1A3	PANC-1 NFATC1 control	48,519	57,592	**69.4**
		PANC-1 NFATC1 KO	33,268	39,951	
	GAPDH	PANC-1 NFATC1 control	84,246		
		PANC-1 NFATC1 KO	83,271		

Overexpression results:

Analysis	Protein	Signal Intensity [Arbitrary Units]		Normalized to 100,000 Signal Units GAPDH	Ratio % KO/Control
Overexpression (OE)	NFATC1	MiaPaCa-2 NFATC1 control	116,728	92,558	**162.0**
		MiaPaCa-2 NFATC1 OE	208,834	149,989	
	GAPDH	MiaPaCa-2 NFATC1 control	126,114		
		MiaPaCa-2 NFATC1 OE	139,233		
	NFATC1	PANC-1 NFATC1 control	79,659	66,684	**222.4**
		PANC-1 NFATC1 OE	165,253	148,327	
	GAPDH	PANC-1 NFATC1 control	119,457		
		PANC-1 NFATC1 OE	111,411		
Overexpression (OE)	NFATC1	MiaPaCa-2 NFATC1 control	75,027	58,992	**270.1**
		MiaPaCa-2 NFATC1 OE	179,665	159,337	
	GAPDH	MiaPaCa-2 NFATC1 control	127,182		
		MiaPaCa-2 NFATC1 OE	112,758		
	NFATC1	PANC-1 NFATC1 control	112,068	149,616	**138.6**
		PANC-1 NFATC1 OE	167,077	207,456	
	GAPDH	PANC-1 NFATC1 control	74,904		
		PANC-1 NFATC1 OE	80,536		
Overexpression (OE)	ALDH1A3	MiaPaCa-2 NFATC1 control	26,338	22,048	**133.1**
		MiaPaCa-2 NFATC1 OE	32,687	29,339	
	GAPDH	MiaPaCa-2 NFATC1 control	119,457		
		MiaPaCa-2 NFATC1 OE	111,411		
	ALDH1A3	PANC-1 NFATC1 control	20,997	28,032	**234.6**
		PANC-1 NFATC1 OE	52,970	65,772	
	GAPDH	PANC-1 NFATC1 control	74,904		
		PANC-1 NFATC1 OE	80,536		

Cropped images of Western blot results:

Fig. 3A

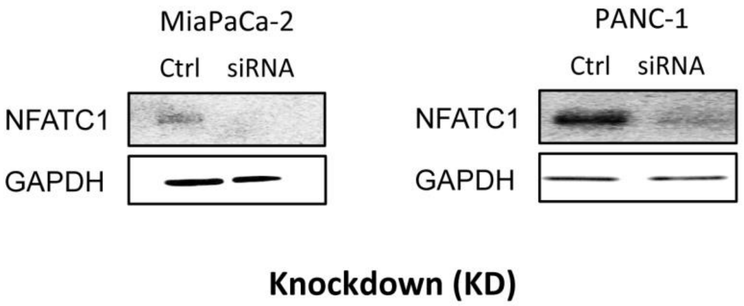

Figs. 3D and 4

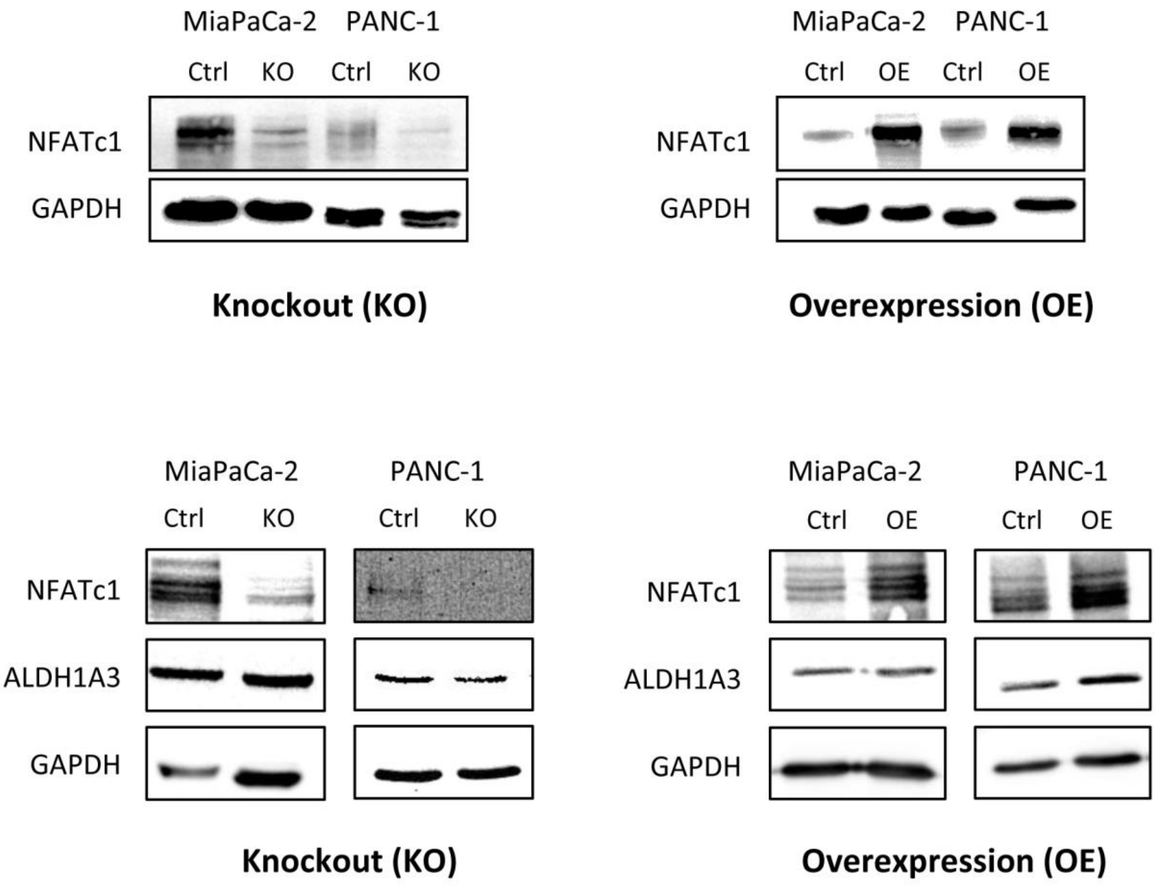

Uncropped images of Western blot results:

KO – MiaPaCa-2:

Full blot

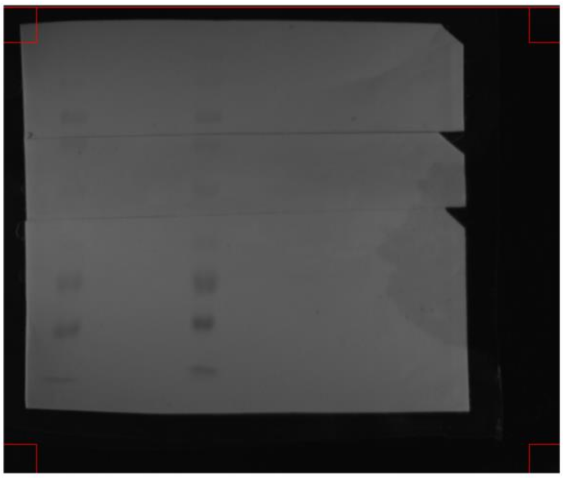

NFATc1

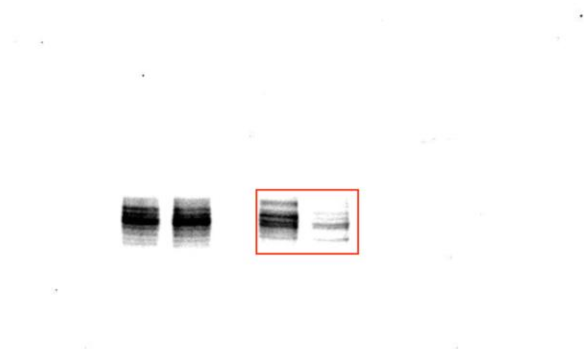

ALDH1A3

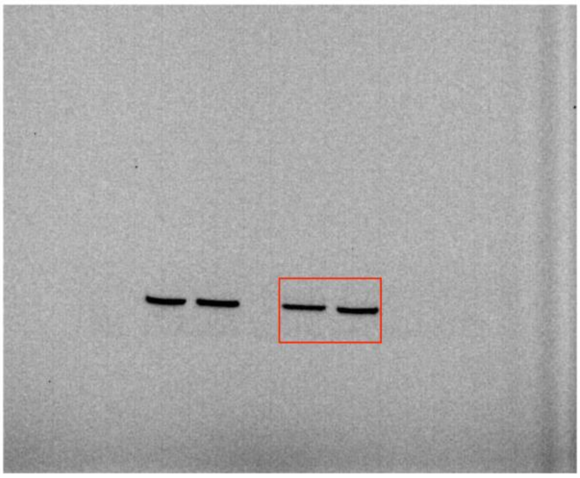

GAPDH

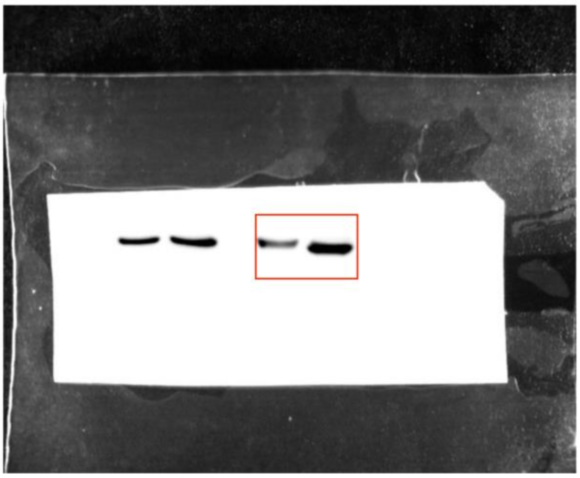

KO – PANC-1:

Full blot

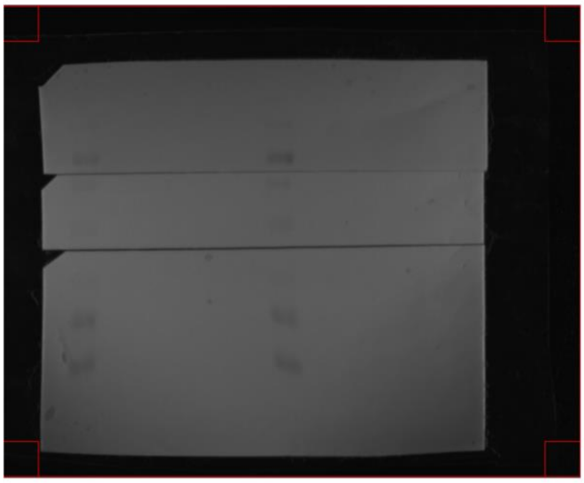

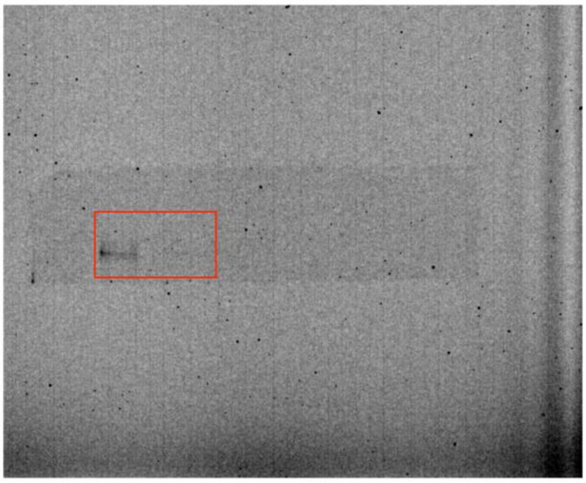

ALDH1A3

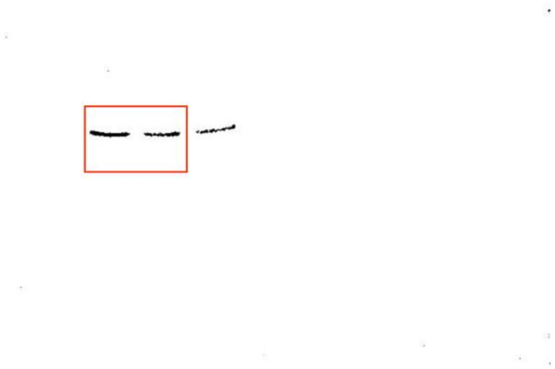

GAPDH

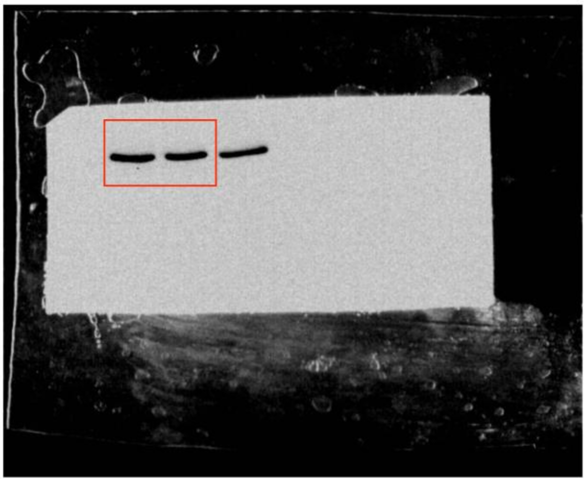

OE – MiaPaCa-2 & PANC-1:

Full blot

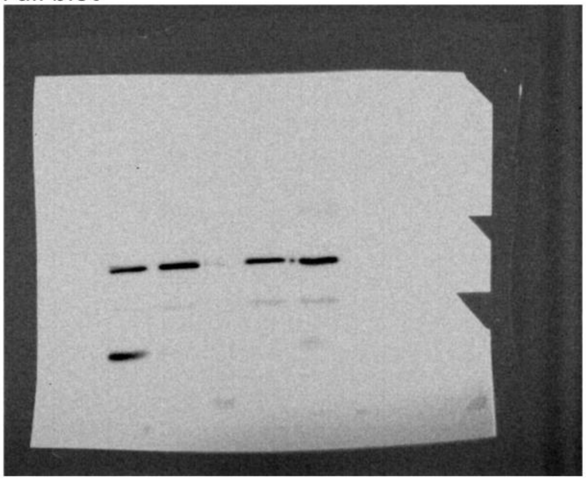

NFATc1

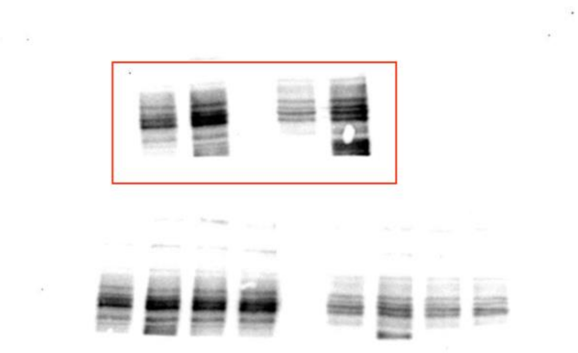

ALDH1A3

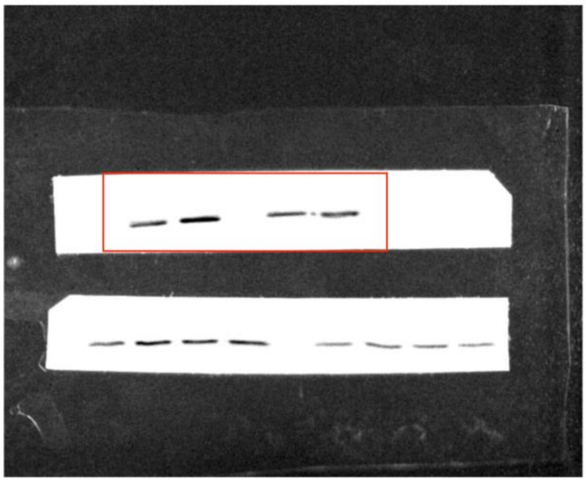

GAPDH

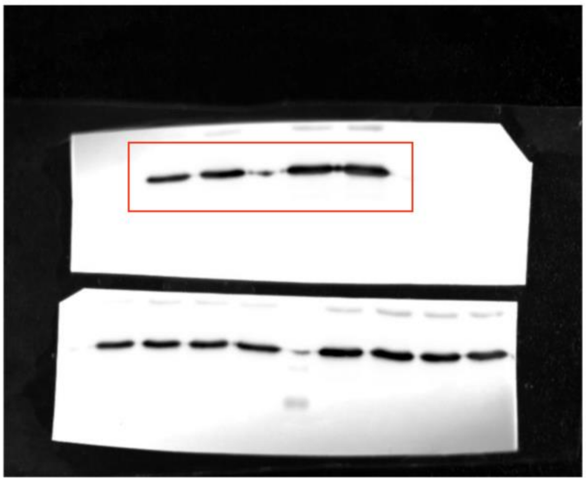

Only the top part in each image is relevant to this paper. The bottom part is an analysis done with other antibodies for another project.

siRNA KD – MiaPaCa-2 & PANC-1:

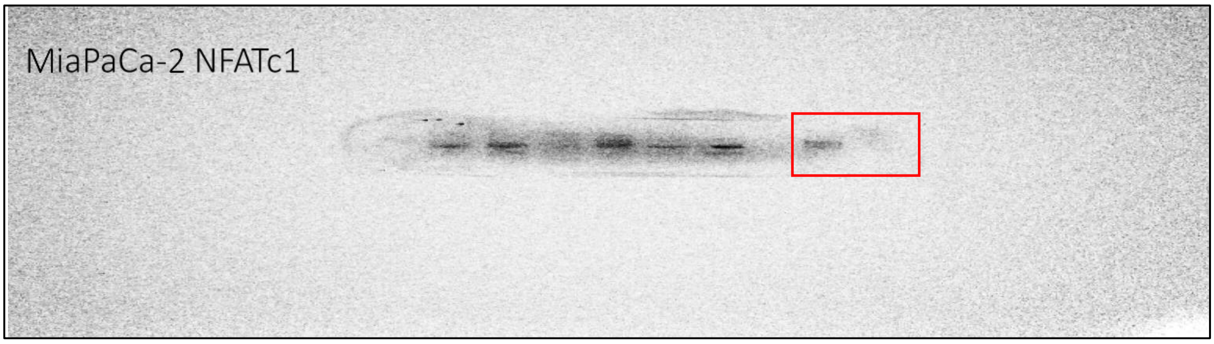

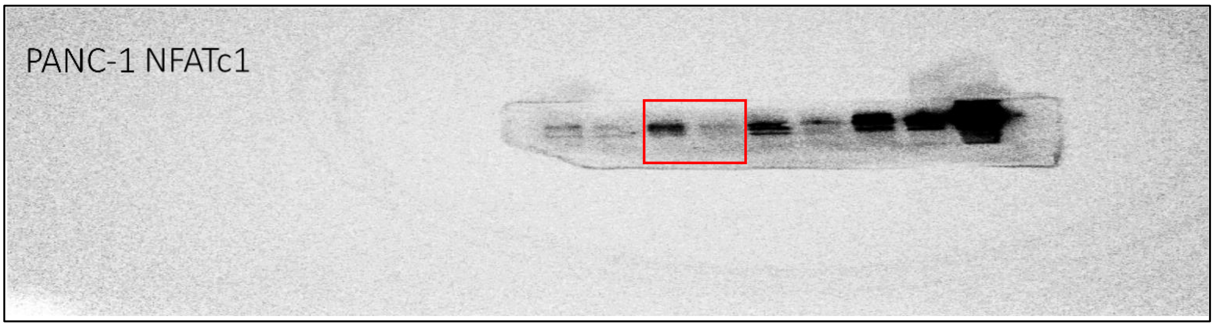

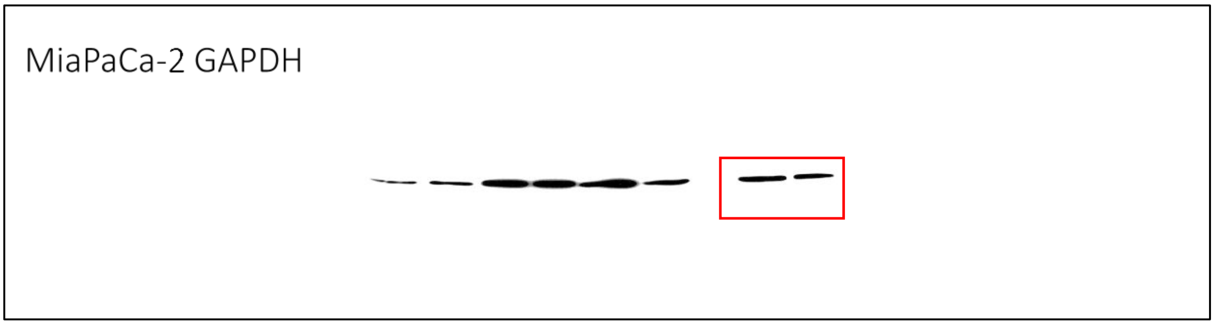

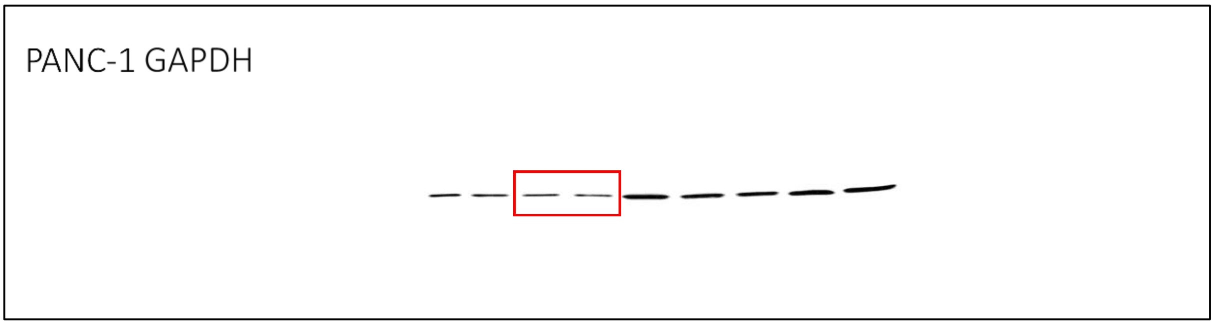

KO – MiaPaCa-2 & PANC-1:

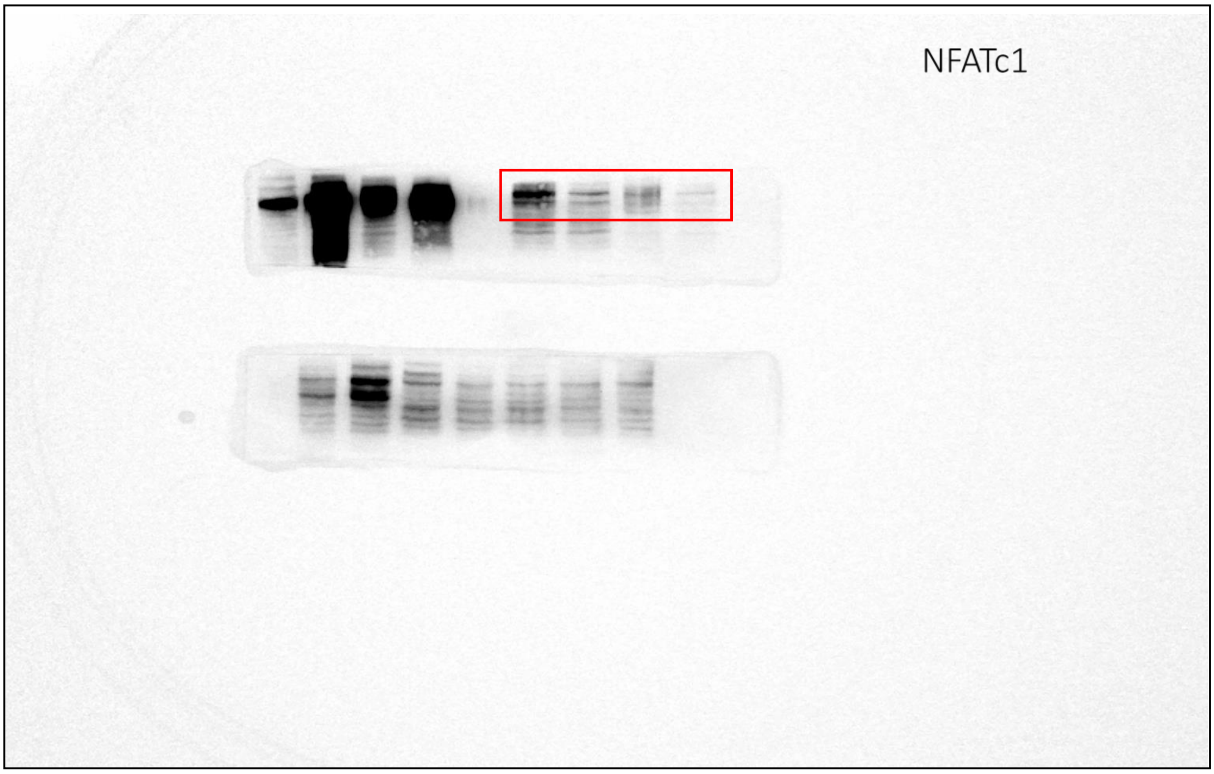

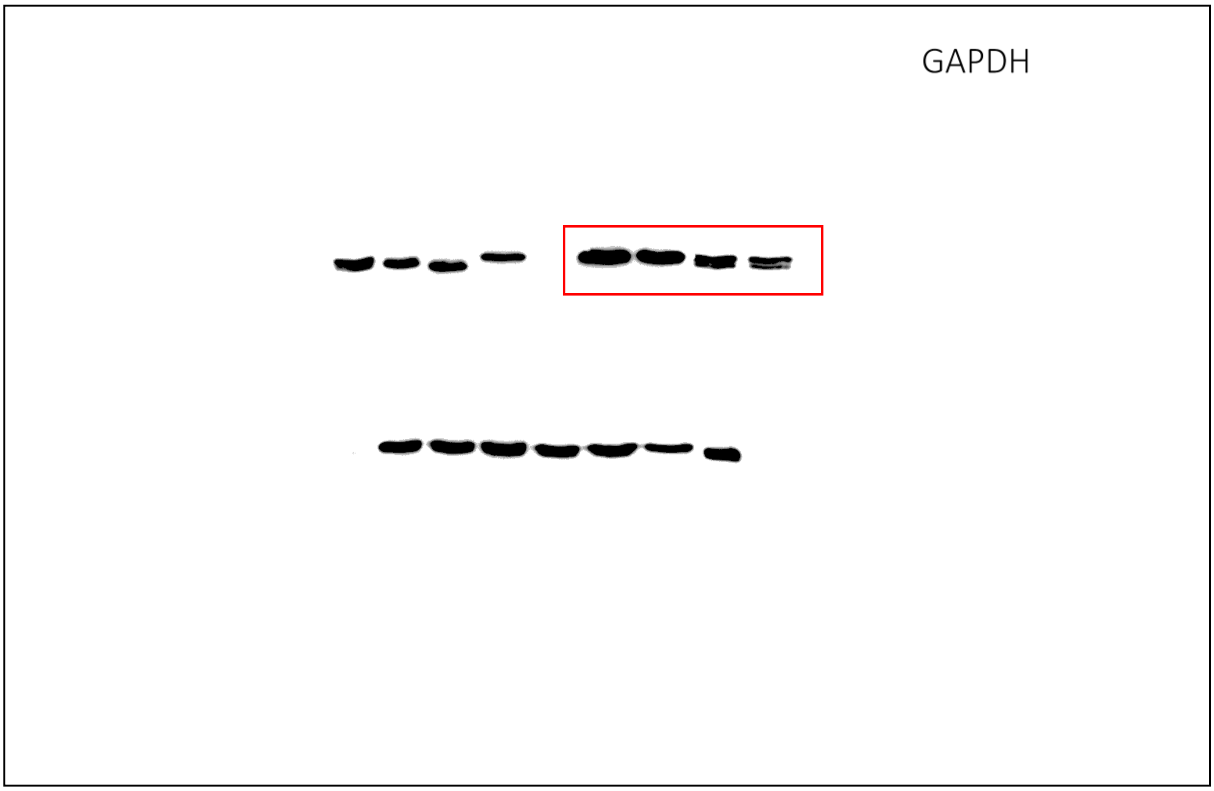

OE – MiaPaCa-2 & PANC-1:

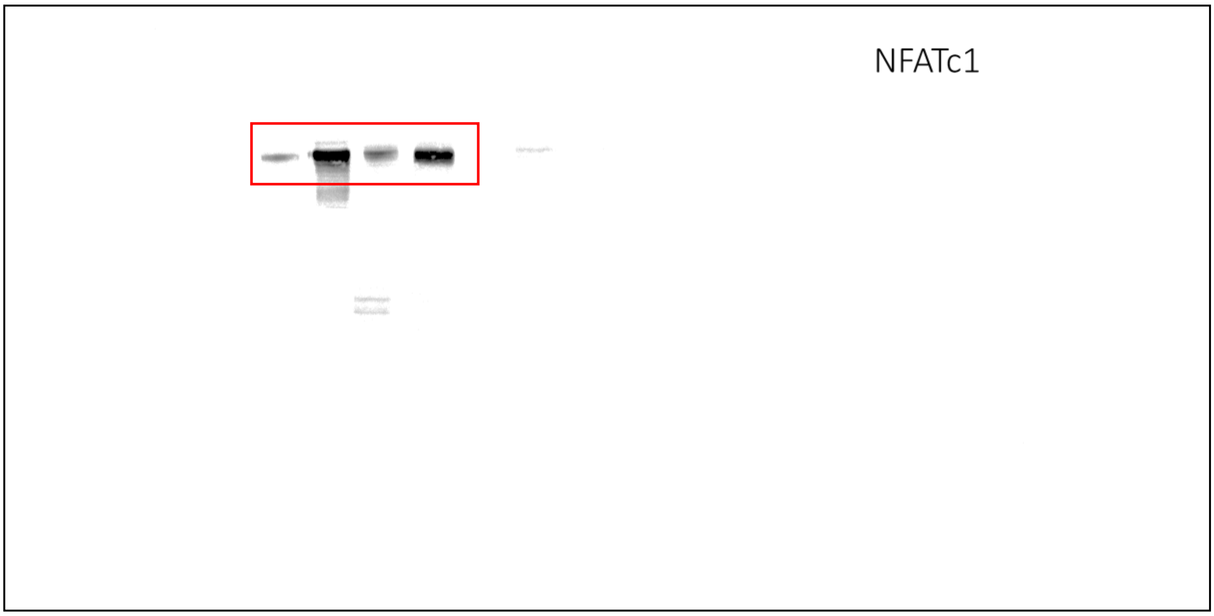

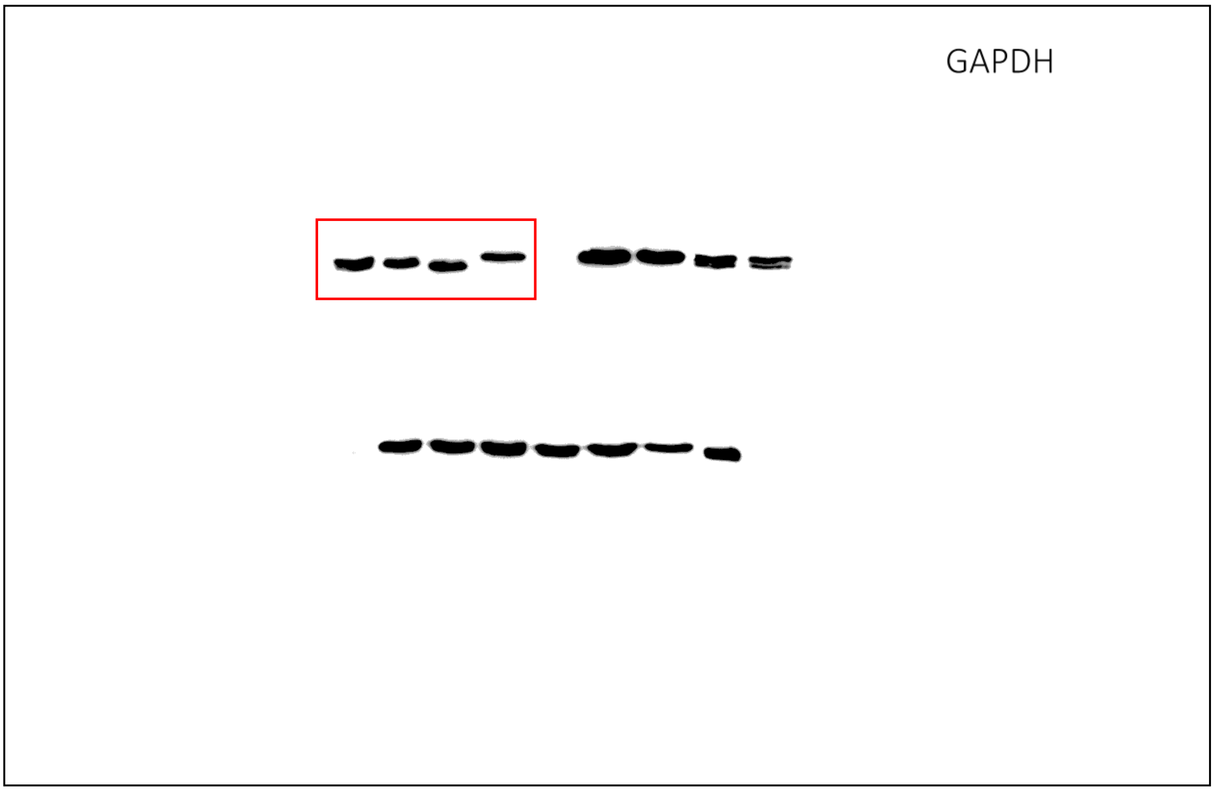

Supplement: Supplementary file 1 [file cancers-13-04569-s001.zip › Supplementary Table S4.pdf]
